# Multidimensional dietary assessment and interpretable machine learning models predict the risk of prediabetes/diabetes and osteoporosis comorbidity in older adults

**DOI:** 10.3389/fnut.2025.1666477

**Published:** 2025-11-17

**Authors:** Yuwen ShangGuan, Kangkang Ji, Zhenhao Lin, Chenyiyi He, Young-Je Sim, Haobiao Liu, Kunyi Huang, Kunpeng Wu, Litao Yan, Kunyuan Xu, Huan Li

**Affiliations:** 1 Department of Exercise Physiology, Kunsan National University, Gunsan, Republic of Korea; 2 Department of Clinical Medical Research, Binhai County People’s Hospital, Binhai Clinical College, Yangzhou University Medical College, Yancheng, Jiangsu, China; 3 College of Biomedicine and Health, Huazhong Agricultural University, Wuhan, Hubei, China; 4 Department of Rehabilitation Medicine, Shantou University Medical College/Shenzhen Children's Hospital, Shantou, Guangdong, China; 5 Department of Epidemiology and Biostatistics, School of Public Health, Health Science Center, Xi'an Jiaotong University, Xi'an, Shaanxi, China; 6 Department of Health and Physical Education, The Education University of Hong Kong, Tai Po, Hong Kong SAR, China; 7 Department of Articular Orthopedics, The First People’s Hospital of Changzhou, The Third Affiliated Hospital of Soochow University, Changzhou, China; 8 Department of Endocrinology, Guang’anmen Hospital, China Academy of Chinese Medical Sciences, Beijing, China

**Keywords:** machine learning, diabetes mellitus, osteoporosis, comorbidity, dietary nutrient intake, SHAP analysis, older adults

## Abstract

**Background:**

The health burden of diabetes mellitus and osteoporosis (DM-OP) comorbidity in the aging population is increasing, and dietary factors are modifiable risk determinants. This study developed and validated a machine learning model to predict DM-OP comorbidity using multidimensional dietary assessment.

**Methods:**

This study utilized data from NHANES cycles 2005–2010, 2013–2014, and 2017–2020, ultimately including 4,678 participants aged ≥65 years. Dietary data were collected through 24-h dietary recalls, encompassing macronutrients, micronutrients, food processing classification (NOVA), and five dietary quality scores. Missing data were handled using random forest algorithm, feature selection was performed using Boruta algorithm, and SMOTE technique addressed class imbalance. Eight machine learning algorithms (XGBoost, decision tree, logistic regression, multilayer perceptron, naive Bayes, k-nearest neighbors, random forest, and support vector machine) were implemented with 10-fold cross-validation for performance evaluation.

**Results:**

A total of 4,678 participants were included, with 347 (7.4%) having DM-OP comorbidity (concurrent prediabetes/diabetes and osteoporosis). After feature selection, 46 variables were retained for model construction. The random forest model demonstrated superior predictive performance with the lowest error rate (0.161), highest accuracy (0.839), ROC AUC of 0.965, sensitivity of 0.827, and specificity of 0.852. SHAP analysis revealed gender as the most important predictor, with females at higher risk; BMI showed positive correlation with comorbidity risk; while carotenoid, vitamin E, magnesium, and zinc intake were negatively correlated with disease risk, suggesting potential protective associations. An online risk prediction tool was developed based on the optimized random forest model for real-time individual comorbidity risk calculation.

**Conclusion:**

The random forest model demonstrated excellent performance in predicting diabetes-osteoporosis comorbidity in elderly adults, with gender, BMI, and specific nutrient intake as key predictors. This model provides an effective tool for clinical early identification of high-risk populations and implementation of preventive interventions.

## Graphical abstract

**Figure:**
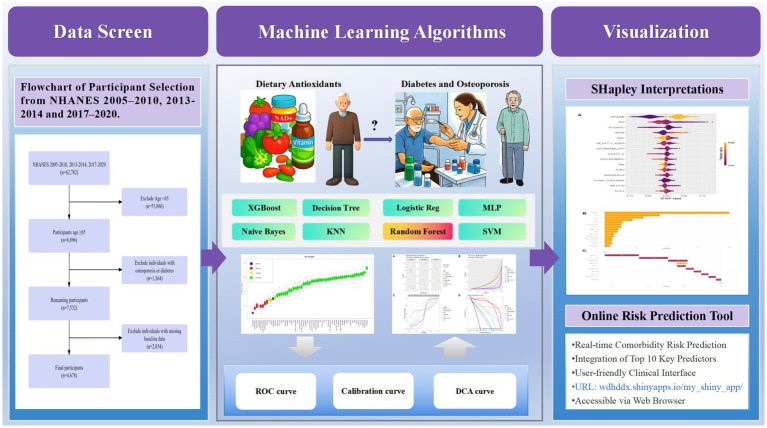

## 1 Introduction

The global burden of chronic diseases has significantly increased, with diabetes mellitus (DM) and osteoporosis (OP) becoming intertwined public health challenges, especially among older adults (1). DM, characterized by persistent high blood sugar levels, currently affects over 537 million adults worldwide, with projections estimating 783 million by 2045 (2). Type 2 diabetes mellitus (T2DM) accounts for 90–95% of these cases and is often associated with obesity, physical inactivity, and aging (3, 4). Conversely, osteoporosis, defined by low bone mineral density and deteriorating bone structure, affects approximately 200 million people globally (5), with hip fractures resulting in a 20% mortality rate within one year (6). Individuals with DM face a 1.5- to 2-fold higher risk of developing osteoporosis and fractures (7), primarily due to oxidative stress from high blood sugar levels, insulin resistance, and disrupted bone remodeling (8, 9). Additionally, prediabetes, affecting 374 million adults worldwide, offers a critical window for intervention, with a 35–50% chance of progressing to T2DM within 5–10 years (3). Recent studies establish a connection between prediabetes and early bone loss (10, 11), though the underlying mechanisms remain poorly understood (12). This lack of clarity underscores the importance of exploring prediabetes as a potential precursor to osteoporosis, particularly in aging populations.

Dietary factors play a pivotal role in the pathogenesis of both diabetes and osteoporosis. Diets rich in pro-inflammatory components, such as trans fats and refined carbohydrates, exacerbate systemic inflammation, thereby promoting insulin resistance and accelerating bone loss (13). Conversely, foods high in antioxidants, such as fruits and vegetables, mitigate oxidative stress, potentially offering protective effects against both conditions (14). Ultra-processed foods (UPFs), characterized by high additive content and low nutritional value, are strongly associated with increased risks of both diseases due to their induction of inflammation and metabolic disturbances (15, 16). Understanding the roles of these dietary factors is critical for developing effective prevention strategies. Furthermore, the impact of overall dietary patterns on bone health is evident across diverse populations, as demonstrated by a study linking maternal diet to bone density in Chinese lactating women and their infants (17). However, most studies have focused on the effects of individual nutrients, and there remains a lack of systematic exploration into how dietary patterns or composite dietary factors influence the combined pathogenesis of diabetes and osteoporosis.

In this context, machine learning algorithms have emerged as powerful tools for disease prediction, capable of analyzing complex, multidimensional data to create effective predictive models (18, 19). This study uses data from the National Health and Nutrition Examination Survey (NHANES) database, employing eight machine learning algorithms and SHAP analysis. The goal is to investigate the predictive role of dietary antioxidants for the comorbidity of prediabetes or diabetes with osteoporosis in older adults, identify key contributing factors, and develop an online prediction tool to provide a scientific basis and practical tool for early detection and intervention.

## 2 Methods

### 2.1 Data source and study population

**Figure 1:**
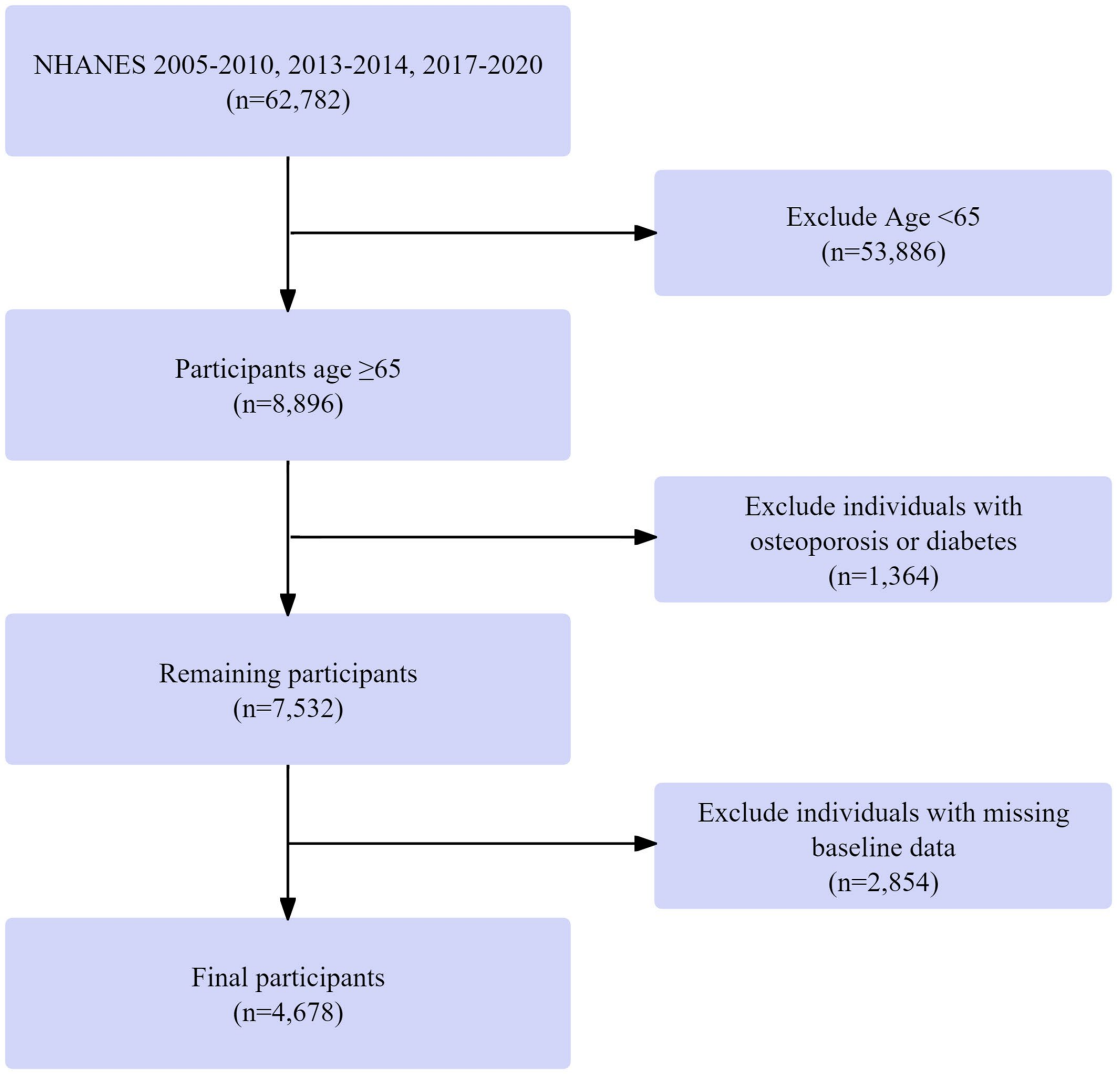
Flowchart of participant selection in the NHANES dataset.

Data were drawn from the 2005–2010, 2013–2014, and 2017–2020 NHANES cycles, including 62,782 individuals. These particular cycles were chosen because they encompass all available recent NHANES data cycles that contain both dual-energy X-ray absorptiometry (DXA) measurements for bone mineral density assessment and the detailed dietary data from 24-h recalls required for our multidimensional dietary assessment. Those under 65 years were excluded (53,886 removed), leaving 8,896 participants aged ≥ 65. Next, 1,364 individuals with missing diabetes or osteoporosis data were excluded, resulting in 7,532 participants. Finally, 2,854 individuals with missing baseline data were removed, yielding a final cohort of 4,678 participants with complete data on age, diabetes/osteoporosis status, and baseline measurements. The screening process is shown in Figure 1.

### 2.2 Dietary assessment

Dietary data were collected via two 24-h dietary recall interviews. Macronutrient intake included total carbohydrates, protein, total fat, saturated fat, monounsaturated fatty acids (MUFA), polyunsaturated fatty acids (PUFA), omega-3 fatty acids, omega-6 fatty acids, and cholesterol. Micronutrient assessment included vitamins (A, C, D, E, B6, B12, riboflavin, niacin, thiamin, folate), minerals (calcium, magnesium, iron, zinc, copper, selenium), and phytochemicals (alpha-carotene, beta-carotene, carotene retinol equivalents). Dietary fiber and caffeine intake were quantified. The intake data for macronutrients, micronutrients, and other dietary components were sourced directly from the NHANES-provided Total Nutrient Intake files (e.g., DR1TOT_J and DR2TOT_J). The conversion of food items reported in the 24-h recalls to quantitative nutrient values was performed by the National Center for Health Statistics (NCHS) using the USDA’s Food and Nutrient Database for Dietary Studies (FNDDS), which links each food to its detailed nutritional composition (20). All nutrient intakes were energy-adjusted using the residual method. The average intake from two non-consecutive 24-h recalls was used as a proxy for usual intake, as this method reduces within-person variability and provides a more stable estimate of habitual consumption, consistent with established dietary assessment methodology.

#### 2.2.1 Food processing classification

Food items were categorized into NOVA groups based on established classification rules using USDA food codes. To ensure coding consistency, a random subset of food items underwent dual coding by independent researchers, with discrepancies resolved through adjudication. Food items were categorized using the NOVA classification system into unprocessed and minimally processed foods, processed culinary ingredients, processed foods, and ultra-processed foods, with daily consumption calculated in grams. For specific coding rules for classification and to access the classification consistency assessment, please refer to Supplementary Table S2.

#### 2.2.2 Dietary quality indices

Five validated dietary quality scores were calculated: Composite Dietary Antioxidant Index (CDAI), Dietary Inflammatory Index (DII), Healthy Eating Index 2020 (HEI-2020), Dietary Approaches to Stop Hypertension (DASH) score, and Oxidative Balance Score (OBS). Detailed calculation methods are provided in Supplementary material S1. All dietary variables were energy-adjusted and averaged across the two 24-h recalls to reflect usual intake. The use of two non-consecutive 24-h recalls, as implemented in NHANES, is a well-established method for estimating usual dietary intake at the population level. Averaging intake from 2 days helps to attenuate within-person day-to-day variation and provides a more stable estimate of habitual consumption than a single day’s recall. Furthermore, the dietary quality indices used (CDAI, DII, HEI-2020, DASH, OBS) are composite measures that are less sensitive to daily fluctuations and more representative of longer-term dietary patterns.

### 2.3 Diagnosis of diabetes and osteoporosis

Diabetes was diagnosed using multiple criteria: physician diagnosis, fasting blood glucose ≥7.0 mmol/L, or glycosylated hemoglobin (HbA1c) ≥ 6.5%. Prediabetes was defined as fasting blood glucose of 5.6–6.9 mmol/L or HbA1c of 5.7–6.4%. The use of HbA1c and FPG, consistent with contemporary ADA guidelines and the operational feasibility of the NHANES protocol, provides a standardized approach for identifying dysglycemia in this large cohort, though it may not capture individuals with isolated postprandial hyperglycemia. Osteoporosis was diagnosed using standard dual-energy X-ray absorptiometry (DXA). The DXA examinations were performed using Hologic QDR-4500A fan-beam densitometers (Hologic, Inc., Bedford, Massachusetts) following the standardized NHANES body composition procedures manual. The scans were analyzed using Hologic APEX 4.0 software. A T-score of ≤ − 2.5 at the femoral neck was used to define osteoporosis, For detailed diagnostic criteria, refer to Supplementary Table S3. Furthermore, it should be noted that the application of the uniform T-score threshold (≤ − 2.5) may have limitations across different sex and racial/ethnic groups, as population-specific bone mineral density reference data and fracture risk relationships can vary.

We define the “DM-OP” group as the “Diabetes Mellitus and Diabetes-Osteoporosis Comorbidity Group.” This group comprises participants who meet both of the following criteria: having osteoporosis and concurrently having diabetes mellitus or prediabetes. The non–DM-OP group included all participants without this comorbidity. Additionally, we conducted a sensitivity analysis by training separate interpretable machine learning models using only DM and only OP outcomes to distinguish between disease-specific dietary and metabolic predictors.

### 2.4 Covariates

Covariates included demographic, socioeconomic, anthropometric, clinical, and lifestyle factors. Demographic and socioeconomic variables comprised age (continuous), gender (male/female), race/ethnicity (Non-Hispanic White, Non-Hispanic Black, Mexican American, Other Hispanic, Other Race), marital status (married/living with partner, widowed/divorced/separated, never married), and educational attainment (below high school, high school, above high school). Socioeconomic status was assessed using the PIR, categorized as <1, 1–3, and >3. Anthropometric and clinical measurements included BMI, calculated from measured height and weight, and the presence of physician-diagnosed or laboratory-confirmed conditions such as hypertension, hyperlipidemia, cardiovascular disease, and stroke. Glucose metabolism abnormalities were defined as impaired fasting glycemia (fasting glucose 5.6–6.9 mmol/L) and impaired glucose tolerance (2-h glucose 7.8–11.0 mmol/L based on oral glucose tolerance testing). Serum 25-hydroxyvitamin D [25(OH)D] concentrations were determined using standardized laboratory assays. Lifestyle and behavioral factors encompassed smoking status (never, former, current), alcohol consumption (grams per day estimated from 24-h dietary recalls), and serum cotinine concentration, which served as an objective biomarker of tobacco exposure.

### 2.5 Preprocessing of machine learning features

The initial dataset included 56 variables: 13 categorical and 43 continuous. Missing data were imputed using a random forest (RF) algorithm with 100 trees and up to 10 iterations. Imputation quality was evaluated using normalized root mean square error (NRMSE) for continuous variables and proportion of falsely classified (PFC) for categorical variables, both indicating reliable performance. Feature selection was performed using the Boruta algorithm to identify variables most relevant to the target outcome. To address class imbalance, we applied the Synthetic Minority Oversampling Technique (SMOTE), which increased the number of comorbid cases from 347 to 3,817, resulting in a total dataset of 4,331 participants (see Supplementary Figure S1 for details). To prevent data leakage, all preprocessing steps—including data imputation, feature selection, and SMOTE oversampling—were strictly conducted within the training folds during cross-validation.

### 2.6 Statistical analysis

Eight machine learning algorithms were implemented using the mlr3 framework: XGBoost, decision tree, logistic regression (LR), multilayer perceptron (MLP), naive Bayes, k-nearest neighbors (KNN), RF, and support vector machine with radial basis function (SVM-RBF). These algorithms were selected for their complementary strengths and proven effectiveness in medical prediction tasks (21–23). Model performance was assessed using classification error rate, accuracy, F-beta score, area under the ROC curve (AUC), sensitivity, specificity, and area under the precision-recall curve (AUPRC). Ten-fold cross-validation ensured robust performance estimation. Statistical significance of performance differences between models was evaluated using analysis of variance (ANOVA) and the Kruskal-Wallis H test. Feature importance in the optimal model was quantified using SHAP (SHapley Additive exPlanations) values, which provide global feature rankings and local explanations for individual predictions based on game theory. All analyses were conducted in R (version 4.4.3) using the packages survey, DMwR, ggcor, mlr3, mlr3benchmark, mlr3extralearner, kernelshap, and shapviz. Statistical significance was set at *p* < 0.05 for all tests.

## 3 Results

### 3.1 Baseline characteristics of the population

A total of 4,678 participants were included in the analysis: 4,331 (92.6%) in the non-DM-OP (i.e., participants without the coexistence) group and 347 (7.4%) in the DM-OP comorbidity group. The DM-OP group was significantly older and predominantly female (*p* < 0.001). They exhibited lower socioeconomic status, with higher poverty rates and lower educational attainment (p < 0.001). Although the DM-OP group had a lower body mass index (BMI), they showed a higher prevalence of diabetes mellitus (p < 0.001) and impaired glucose metabolism. Their dietary intake included significantly less total fat, protein, and multiple micronutrients, with lower CDAI scores (*p* = 0.031) and higher DII scores (*p* = 0.007), as shown in Table 1.

**Table 1:** Baseline characteristics of the study population according to DM-OP comorbidity status.

Variable	Non-DM-OP (*n*=4331)	DM-OP (*n*=347)	*p*-value
Age (year), mean ± SD	73.02 ± 5.41	74.98 ± 5.36	<0.001***
Gender, *n* (%)			<0.001***
Female	1959 (45.2)	267 (76.9)	
Male	2372 (54.8)	80 (23.1)	
Race, *n* (%)			<0.001***
Non-Hispanic White	2530 (58.4)	190 (54.8)	
Non-Hispanic Black	834 (19.3)	23 (6.6)	
Mexican American	396 (9.1)	53 (15.3)	
Other Hispanic	299 (6.9)	47 (13.5)	
Other Race	272 (6.3)	34 (9.8)	
Marital status, *n* (%)			<0.001***
Married/Living with Partner	2574 (59.4)	146 (42.1)	
Widowed/Divorced/Separated	1610 (37.2)	183 (52.7)	
Never married	147 (3.4)	18 (5.2)	
PIR, *n* (%)			<0.001***
<1	1047 (24.2)	125 (36.0)	
1-3	1982 (45.8)	163 (47.0)	
>3	1302 (30.1)	59 (17.0)	
Education level, *n* (%)			<0.001***
Below high school	577 (13.3)	90 (25.9)	
High school	1663 (38.4)	149 (42.9)	
Above high school	2091 (48.3)	108 (31.1)	
Smoking status, *n* (%)			<0.001***
Never	2033 (46.9)	207 (59.7)	
Former	1890 (43.6)	108 (31.1)	
Current	408 (9.4)	32 (9.2)	
BMI (kg/m^2^), mean ± SD	29.01 ± 5.31	27.16 ± 5.21	<0.001***
Hypertension, yes, *n* (%)	3207 (74.0)	264 (76.1)	0.442
Hyperlipidemia, yes, *n* (%)	3639 (84.0)	298 (85.9)	0.404
Impaired fasting glycaemia, yes, *n* (%)	312 (7.2)	55 (15.9)	<0.001***
Impaired glucose tolerance, yes, *n* (%)	213 (4.9)	62 (17.9)	<0.001***
Diabetes mellitus, yes, *n* (%)	1575 (36.4)	230 (66.3)	<0.001***
Osteoporosis, yes, *n* (%)	392 (9.1)	347 (100.0)	<0.001***
Cardiovascular disease, yes, *n* (%)	1214 (28.0)	100 (28.8)	0.801
Stroke, yes, *n* (%)	410 (9.5)	44 (12.7)	0.064
25-OH-D (nmol/L), mean ± SD	74.82 ± 26.72	71.57 ± 27.58	0.030*
Dietary fiber (g/day), mean ± SD	16.77 ± 7.85	15.46 ± 5.85	0.002**
Total fat (g/day), mean ± SD	72.09 ± 31.08	61.56 ± 26.88	<0.001***
Alpha-carotene (μg/day), mean ± SD	462.94 ± 870.72	467.66 ± 613.18	0.921
Beta-carotene (μg/day), mean ± SD	2484.25 ± 2901.38	2406.55 ± 2619.22	0.629
Riboflavin (mg/day), mean ± SD	1.97 ± 0.81	1.79 ± 0.72	<0.001***
Niacin (mg/day), mean ± SD	22.03 ± 9.29	19.89 ± 8.24	<0.001***
Vitamin B6 (mg/day), mean ± SD	1.91 ± 0.94	1.78 ± 1.06	0.012*
Folate (μg/day), mean ± SD	367.55 ± 166.07	341.86 ± 131.75	0.005**
Vitamin B12 (μg/day), mean ± SD	4.88 ± 5.46	4.30 ± 5.24	0.055
Vitamin C (mg/day), mean ± SD	85.21 ± 63.33	86.59 ± 65.81	0.697
Vitamin E (mg/day), mean ± SD	7.73 ± 4.46	6.45 ± 3.12	<0.001***
Calcium (mg/day), mean ± SD	843.36 ± 380.18	780.70 ± 335.29	0.003**
Magnesium (mg/day), mean ± SD	278.19 ± 107.65	251.49 ± 81.17	<0.001***
Iron (mg/day), mean ± SD	14.34 ± 6.55	12.84 ± 5.10	<0.001***
Zinc (mg/day), mean ± SD	10.45 ± 5.27	9.08 ± 3.59	<0.001***
Copper (mg/day), mean ± SD	1.21 ± 0.84	1.10 ± 0.93	0.015*
Selenium (μg/day), mean ± SD	98.70 ± 41.50	88.72 ± 35.33	<0.001***
Alcohol (g/day), mean ± SD	6.07 ± 14.30	2.99 ± 9.30	<0.001***
Cotinine (ng/mL), mean ± SD	34.33 ± 111.33	32.44 ± 104.88	0.761
Carotene RE (μg/day), mean ± SD	226.49 ± 266.68	221.28 ± 233.08	0.724
Vitamin A (μg RE/day), mean ± SD	691.02 ± 561.08	636.40 ± 563.52	0.081
Vitamin D (μg/day), mean ± SD	4.60 ± 3.08	4.33 ± 2.80	0.117
Caffeine (mg/day), mean ± SD	0.15 ± 0.13	0.12 ± 0.10	<0.001***
Carbohydrates (g/day), mean ± SD	220.03 ± 78.11	203.68 ± 65.82	<0.001***
Cholesterol (mg/day), mean ± SD	257.00 ± 135.61	213.34 ± 116.51	<0.001***
Magnesium (mg/day), mean ± SD	278.70 ± 107.76	252.35 ± 81.67	<0.001***
MUFA (g/day), mean ± SD	25.30 ± 10.64	21.67 ± 9.70	<0.001***
N3 fatty acids (g/day), mean ± SD	0.10 ± 0.30	0.07 ± 0.25	0.041*
N6 fatty acids (g/day), mean ± SD	16.16 (7.80	14.07 (7.53	<0.001***
Protein (g/day), mean ± SD	71.56 ± 26.22	64.83 ± 22.96	<0.001***
PUFA (g/day), mean ± SD	16.16 ± 7.05	14.05 ± 6.74	<0.001***
Saturated fat (g/day), mean ± SD	23.33 ± 10.98	19.93 ± 9.15	<0.001***
Thiamin (mg/day), mean ± SD	1.50 ± 0.60	1.37 ± 0.49	<0.001***
Processed food (g/day), mean ± SD	716.63 ± 533.89	681.23 ± 513.04	0.233
Ultra-processed food (g/day), mean ± SD	816.96 ± 730.55	812.85 ± 696.34	0.919
Unprocessed food (g/day), mean ± SD	1112.45 ± 983.74	1100.07 ± 948.77	0.821
CDAI (score), mean ± SD	0.16 ± 3.23	-0.23 ± 2.96	0.031*
DII (score), mean ± SD	1.23 ± 1.77	1.49 ± 1.60	0.007**
HEI2020 (score), mean ± SD	53.80 ± 10.43	54.68 ± 9.05	0.129
DASH (score), mean ± SD	20.87 ± 6.62	20.84 ± 6.25	0.932
OBS (score), mean ± SD	17.15 ± 8.14	16.29 ± 7.73	0.055

PIR, Poverty Income Ratio; BMI, Body Mass Index; 25-OH-D, 25-hydroxyvitamin D; MUFA, Monounsaturated Fatty Acids; PUFA, Polyunsaturated Fatty Acids; CDAI, Composite Dietary Antioxidant Index; DII, Dietary Inflammatory Index; HEI2020, Healthy Eating Index 2020; DASH, Dietary Approaches to Stop Hypertension; OBS Oxidative Balance Score. *p*< 0.05 *, *p* < 0.01**, *p* < 0.001***.

The DM-OP comorbidity group includes participants with both osteoporosis and dysglycemia (prediabetes or diabetes). The non–DM-OP group includes all participants without the comorbidity (i.e., those with neither condition, with dysglycemia alone, or with osteoporosis alone).

### 3.2 Development and validation of comorbidity prediction models

**Figure 2:**
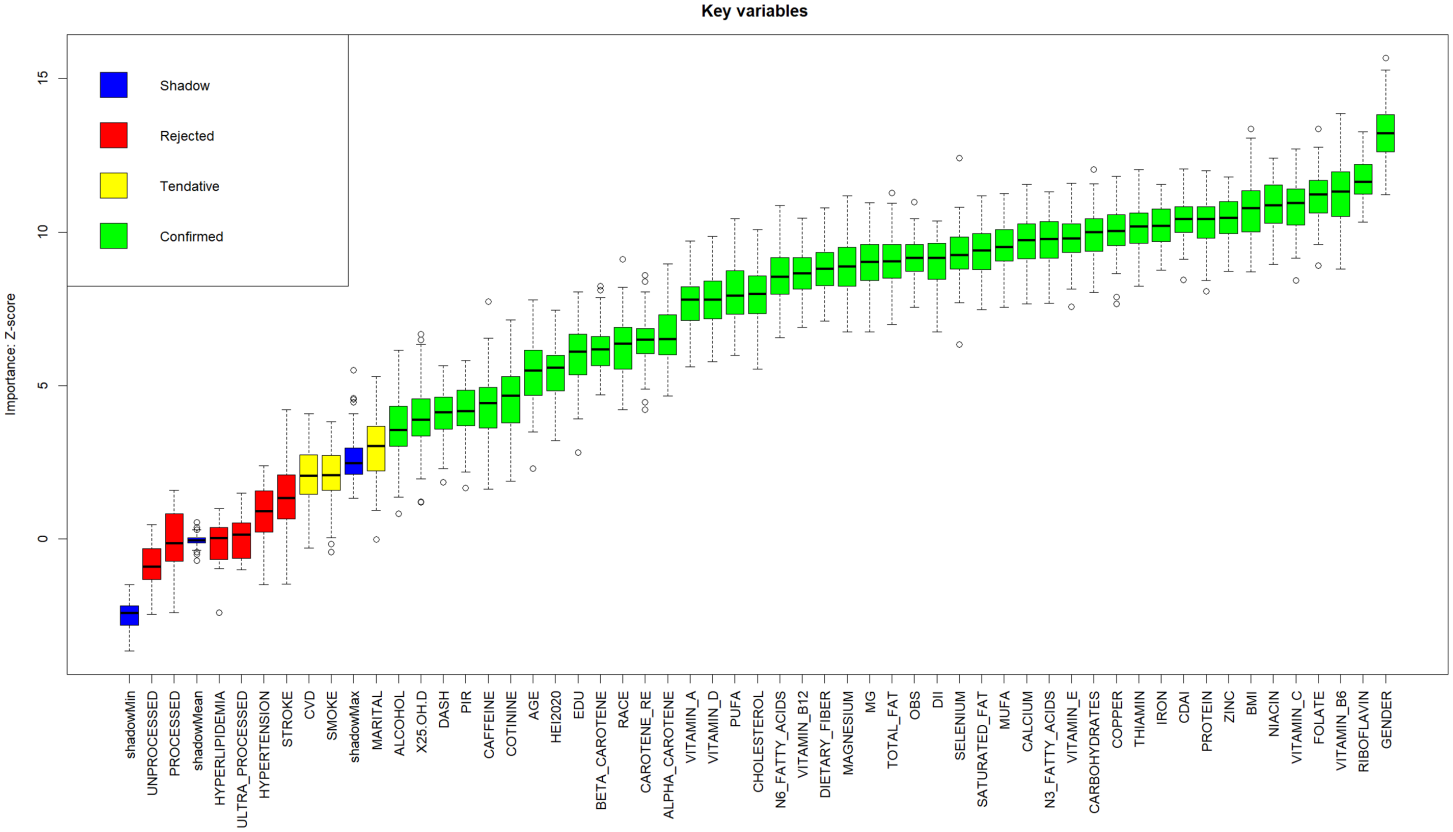
Boxplot of Z-scores for key variable importance. Variables are categorized into four classes (Shadow, Rejected, Tentative, Confirmed) according to their importance Z-scores. Boxplots display statistical summaries: median (horizontal line within boxes), interquartile range (boxes), range (whiskers), and outliers (dots). Higher Z-scores indicate stronger contributions of variables to the model.

Prior to model construction, visual features were generated with categorical feature distributions presented in Supplementary Figure S2 and continuous feature distributions in Supplementary Figure S3. The Boruta algorithm was used to screen out collinearity based on correlation coefficients. As shown in Figure 2, nine variables (hyperlipidemia, hypertension, processed, stroke, ultra-processed, unprocessed, marital status, smoking status, and cardiovascular disease) were excluded, retaining 46 variables for model development.

**Figure 3:**
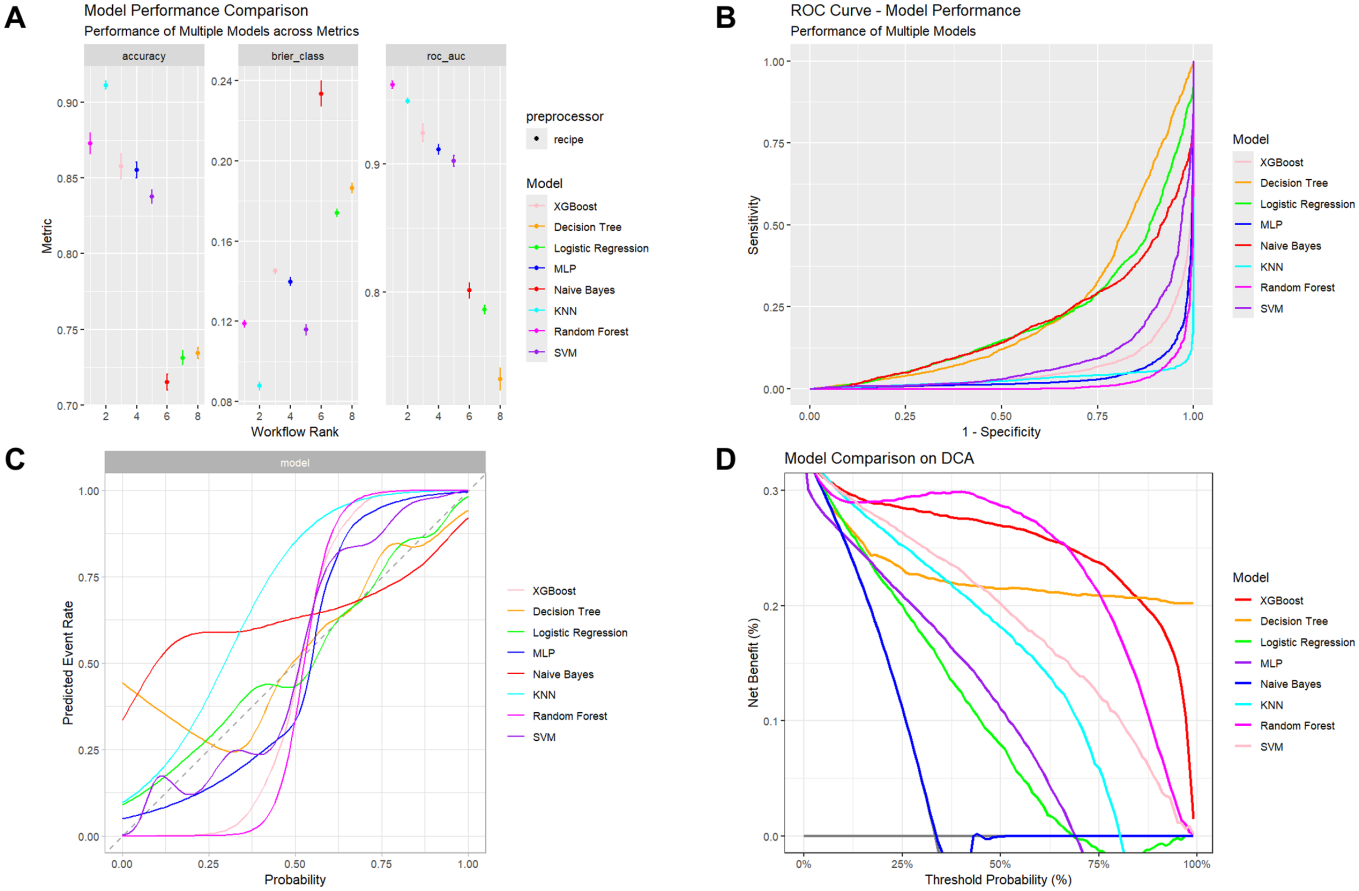
Performance assessment of multiple classifiers. **(A)** Comparison of accuracy, Brier score, and ROC-AUC among models. **(B)** ROC curves showing models’ discrimination performance. **(C)** Calibration curves illustrating agreement between predicted and observed outcomes. **(D)** Decision Curve Analysis (DCA) showing the net clinical benefit across probability thresholds.

Model performance evaluation results are presented in Table 2 and Figure 3, encompassing key metrics including error rate, accuracy, F-beta score, ROC AUC, sensitivity, specificity, and PR AUC. The RF model demonstrated superior performance with the lowest error rate (0.161), highest accuracy (0.839), and excellent performance across F-beta score (0.845), ROC AUC (0.965), sensitivity (0.827), and specificity (0.852). For clinical application, the optimized Random Forest model demonstrated excellent calibration (Brier score = 0.094, calibration intercept = −0.01, slope = 0.99). For screening purposes, a probability threshold of 0.35 is recommended, providing a balanced performance with PPV = 0.65 and NPV = 0.93, making it suitable for identifying high-risk individuals while reliably ruling out the condition. These results indicate that the RF model effectively balances precision and recall while maintaining strong generalization capability, making it optimal for predicting diabetes or prediabetes combined with osteoporosis risk in adults aged 65 and above.

**Table 2:** Performance comparison of different machine learning models.

Model	Error Rate	Accuracy	F-beta	ROC AUC	Sensitivity	Specificity	PR AUC
XGBoost	0.176	0.824	0.840	0.924	0.868	0.774	0.887
DT	0.310	0.690	0.731	0.732	0.791	0.576	0.697
LR	0.297	0.703	0.732	0.787	0.761	0.638	0.737
MLP	0.403	0.597	0.672	0.916	0.775	0.394	0.603
NB	0.329	0.671	0.714	0.802	0.770	0.559	0.682
KNN	0.241	0.759	0.792	0.951	0.864	0.640	0.812
RF	0.161	0.839	0.845	0.965	0.827	0.852	0.907
SVM	0.205	0.795	0.813	0.905	0.840	0.744	0.839
*p*-value	<0.001^a^	<0.001^a^	<0.001^a^	<0.001^b^	<0.001^a^	<0.001^a^	<0.001^a^

XGBoost, extreme gradient boosting tree; DT, Decision Tree; LR, Logistic Regression; MLP, Multilayer Perceptron; NB, Naive Bayes; KNN, k-Nearest Neighbors; RF, Random Forest; SVM-RBF, Support Vector Machine with RBF kernel; ROC AUC, Receiver Operating Characteristic Area Under Curve; PR AUC, Precision-Recall Area Under Curve.

a Statistical tests: ANOVA test.

b Kruskal-Wallis test.

The XGBoost model also exhibited high performance, particularly in ROC AUC (0.924) and F-beta score (0.840), with accuracy of 0.824 and sensitivity of 0.868, demonstrating robust classification capability. However, compared to the RF model, XGBoost showed slightly inferior specificity (0.774), indicating reduced performance in identifying negative samples.

In contrast, decision tree and logistic regression models showed suboptimal performance, particularly in sensitivity and specificity metrics. The decision tree achieved accuracy of 0.690, F-beta score of 0.731, and ROC AUC of 0.732. Although it demonstrated high sensitivity (0.791), its specificity (0.576) was considerably lower than RF and XGBoost models, potentially leading to elevated false positive rates in clinical applications. The logistic regression model performed similarly poorly, with accuracy of 0.703, F-beta score of 0.732, ROC AUC of 0.787, and relatively low sensitivity (0.761) and specificity (0.638), limiting its effectiveness for accurate comorbidity risk prediction.

While MLP and Naive Bayes models showed reasonable ROC AUC performance (0.916 and 0.802, respectively), their F-beta scores and specificity remained inferior to RF and XGBoost models. The MLP model exhibited a pronounced gap between sensitivity (0.775) and specificity (0.394), suggesting potential overfitting. The Naive Bayes model, despite reasonable specificity (0.559), demonstrated low sensitivity (0.770), limiting its practical clinical utility.

### 3.3 SHAP values interpretation

**Figure 4:**
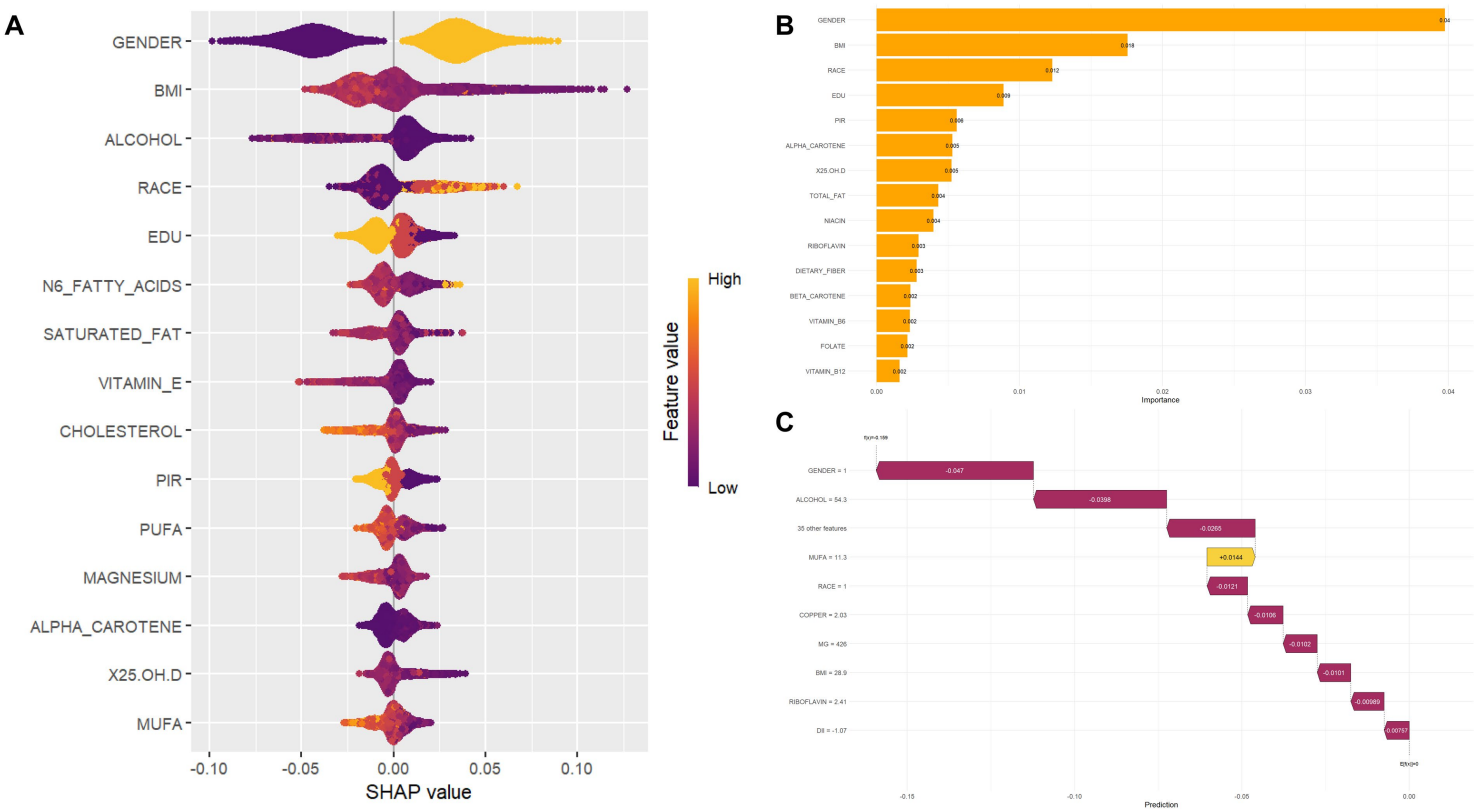
Feature importance and SHAP value interpretation. **(A)** SHAP beeswarm plot visualizing the distribution of SHapley Additive exPlanations (SHAP) values for each feature, colored by feature values (high = orange; low = purple). SHAP values reflect the direction (positive/negative) and magnitude of feature impact on model predictions. **(B)** Bar chart ranking features by their contribution to model performance; taller bars indicate greater influence on predictions. **(C)** SHAP waterfall plot decomposing the cumulative effect of features for a single prediction, where each bar represents the positive (yellow) or negative (purple) contribution of a feature to the final model output.

SHAP analysis (Figure 4) revealed the importance of the top 15 features in predicting comorbid diseases. Gender emerged as a significant predictor of diabetes or prediabetes and osteoporosis comorbidity, with women demonstrating higher susceptibility than men, establishing gender as a crucial factor in disease risk assessment. Additionally, several key features substantially influenced model predictions: BMI, dietary antioxidant intake (particularly carotenoids), vitamin E, magnesium, and zinc. BMI showed positive correlation with DM-OP risk, indicating that higher BMI values correspond to increased comorbidity risk. Conversely, elevated carotenoid intake demonstrated negative correlation with disease risk, suggesting that dietary antioxidants may be associated with protective effects. Against disease development in older adults. High intake of vitamin E and magnesium exhibited suggesting potential protective associations, with magnesium showing particularly strong negative SHAP values, indicating that higher magnesium intake was associated with a lower disease risk. Scatter plots for the top 15 variables are provided in the Supplementary Figures S4, S5.

### 3.4 Sensitivity analysis and stratified analysis

To address potential confounding factors between diabetes and osteoporosis, we performed SHAP analysis on participants with only glucose abnormalities (including prediabetes and diabetes) or only osteoporosis (Supplementary Figures S6, S7). In the diabetes model, BMI and alcohol intake emerged as primary predictors, followed by ethnicity, education, sex, DII, and dietary fiber intake, indicating that obesity levels and nutritional metabolism play a key role in diabetes risk among older adults. Conversely, the osteoporosis model identified sex, BMI, age, niacin intake, and monounsaturated fatty acid intake as primary determinants. Although BMI was present in both models, its SHAP values differed in direction and magnitude, suggesting body composition plays distinct roles in glucose and bone metabolism. These stratified analyses confirm that the risk of diabetes-osteoporosis comorbidity stems from shared metabolic pathways and disease-specific nutritional factors, rather than diagnostic overlap.

**Figure 5:**
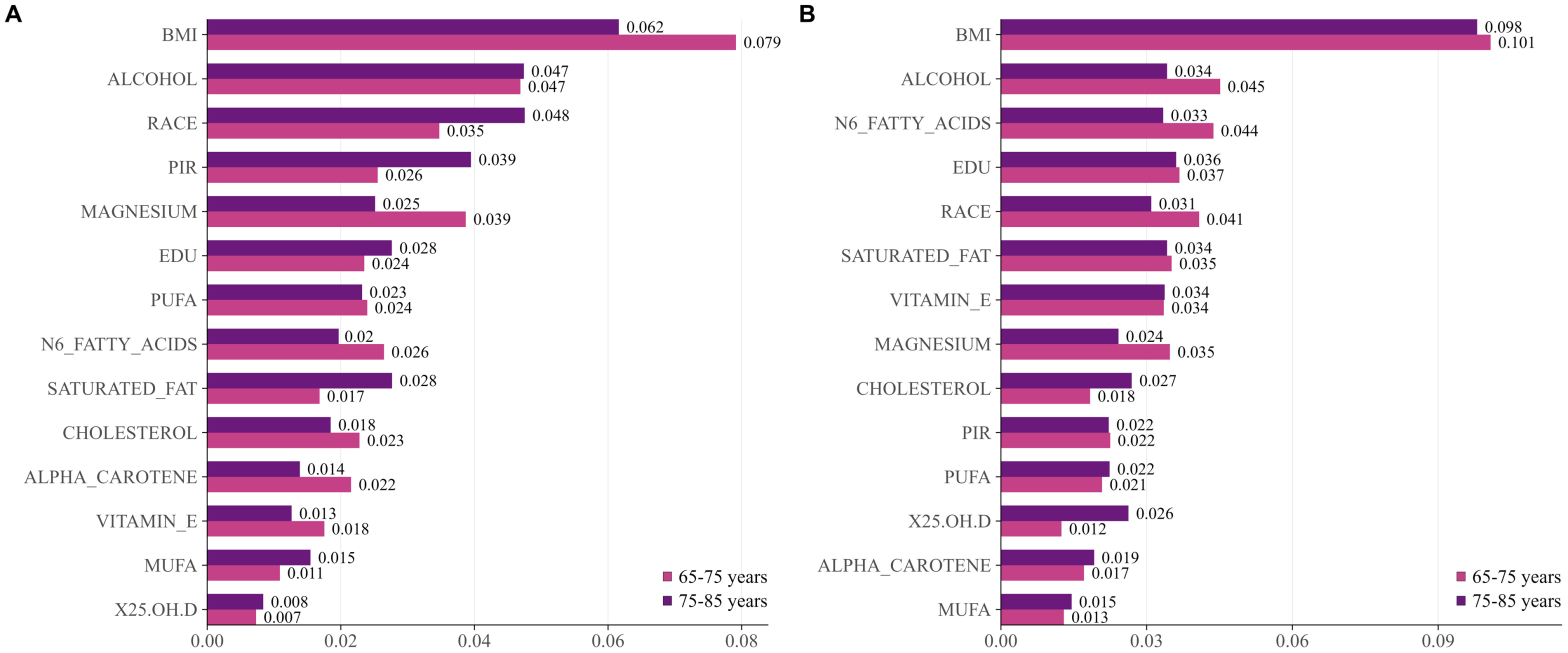
SHAP feature importance under age × gender stratification. **(A)** men; **(B)** women.

Additionally, we conducted an analysis of the “age × gender” interaction term. Figure 5 displays risk factors stratified by gender and age. Women exhibited consistent patterns: BMI was the primary risk factor across all age groups. Men, however, exhibit a differentiated pattern: BMI becomes more prominent among those aged 65–75, while alcohol intake and racial factors exert greater influence in the 75–85 age group. Notably, n-6 fatty acids and educational attainment show greater significance among younger women. Magnesium and vitamin E consistently maintain significant protective effects across all subgroups.

### 3.5 Online prediction tool development and clinical application

Based on the optimized RF model, an online risk prediction tool has been developed, which can be accessed at https://wdhddx.shinyapps.io/osteoporosis-shap-model/. This tool incorporates ten essential indicators, including gender, BMI, and dietary intakes of carotenoids, vitamin E, magnesium, and zinc. It allows for real-time calculations of comorbidity risk probabilities based on individual characteristics entered by the user. The tool is designed with an intuitive interface that seamlessly integrates into clinical workflows, enabling healthcare providers to quickly identify high-risk populations and implement timely interventions aimed at reducing comorbidity risk. This ease of access promotes early detection and supports preventive care strategies within clinical practice.

## 4 Discussion

We utilized interpretable machine learning methods to investigate the relationship between dietary nutrient intake and DM-OP comorbidity in older adults using the U.S. NHANES dataset from 2005 to 2020. Among the 8 machine learning models we evaluated, the RF model stood out with an impressive AUC of 0.965, indicating its excellent predictive capability and stability for classification tasks. Through SHAP game-theoretic analysis, we were able to pinpoint the significance of each selected feature, revealing that gender, BMI, carotenoids, vitamin E, magnesium, and zinc were major contributors to predicting the risk of comorbidity.

To our knowledge, this study is the first to comprehensively develop and validate a prediction model for DM-OP comorbidity that incorporates dietary nutritional factors along with demographic, anthropometric, and clinical characteristics in older adults. While our main focus was on analyzing the role of dietary nutrition, the model also includes easily accessible demographic features, lifestyle factors, and health conditions, which enhances its generalizability. Furthermore, we conducted extensive benchmarking to compare the performance of various machine learning algorithms and created an online prediction tool for clinical use.

Machine learning models are increasingly being used to investigate the dietary and metabolic factors linked to chronic diseases in aging populations. For instance, a recent study comparing five models for predicting all-cause mortality in non-alcoholic fatty liver disease (NAFLD) patients found that Gradient Boosting Machine (GBM) and Random Survival Forest (RSF) achieved strong predictive performance (AUC = 0.8), identifying higher dietary fiber intake as a significant protective factor for improved survival (24). In another study using a Korean cohort, a RF model incorporating genetic polygenic risk scores (gPRS) and metabolite data predicted T2DM occurrence with high accuracy (AUC = 0.883), improving classification by 11.7% over clinical-factor-only models (25). Further demonstrating utility, a CatBoost model integrating dietary antioxidants and metabolic factors effectively predicted chronic kidney disease (CKD) occurrence in individuals with abdominal obesity (AUC = 0.938), identifying age, diabetes history, and dietary antioxidant intake as key predictors (26).

In the realm of DM-OP comorbidity prediction, Wang et al. (27) applied a support vector machine to data from 289 subjects with DM-OP, incorporating five variables: gender, age, BMI, total procollagen I N-terminal propeptide (TP1NP), and osteocalcin, achieving a diagnostic accuracy of 88%. Wu et al. (28) evaluated nine ML algorithms on 303 postmenopausal women with T2DM, with XGBoost showing superior performance (training AUROC: 0.993, testing AUROC: 0.786) using 10 key features, resulting in a clinically applicable risk calculator. Zhao et al. (29) employed RF model to genetically identify hub genes (ACAA2, GATAD2A, VPS35) associated with DM-OP comorbidity. In a separate study of 433 T2DM patients, Wu et al. (30) found XGBoost again performed best (training AUROC: 0.94, testing AUROC: 0.87) among nine ML algorithms for osteoporosis risk prediction, subsequently stratifying patients into high, medium, and low-risk groups using SHAP analysis and Latent Class Analysis (LCA). Yu et al. (31) developed a GBM model using five routine indicators—gender, age, BMI, heart rate, and alkaline phosphatase—in 2,029 individuals with T2DM (457 with osteoporosis), achieving an external validation AUC of 0.89. These studies illustrate the growing application of ML in exploring DM-OP comorbidity prediction, though most focus on clinical indicators such as demographic, anthropometric, and biochemical markers rather than dietary factors.

In this study, we selected 8 diverse ML algorithms (XGBoost, decision tree, logistic regression, MLP, naive Bayes, KNN, RF, and SVM-RBF) to construct prediction models and employed comprehensive benchmarking to determine the optimal approach for diabetes-osteoporosis comorbidity prediction. Compared to traditional statistical methods such as logistic regression, machine learning approaches offer several advantages: they can automatically capture complex non-linear interactions between dietary factors and disease risk without requiring predetermined variable transformations; algorithms like RF and XGBoost can identify the most predictive features through built-in feature importance measures, reducing selection bias; and they demonstrate superior performance in handling high-dimensional healthcare datasets with mixed data types (32).

Our findings suggest that the RF model is the most effective choice for this prediction task. RF is an ensemble method that combines several decision trees, which enhances prediction accuracy through techniques like bootstrap aggregating and random feature selection (33). This model is particularly advantageous in healthcare settings due to its capability to manage various data types, its resistance to overfitting, and its inherent feature importance measures. Additionally, the model’s leaf-wise growth strategy and ensemble characteristics allow it to identify intricate relationships between dietary nutrient intake and comorbidity risk, all while remaining interpretable through SHAP analysis (34). The SHAP analysis indicated that gender, BMI, and certain dietary nutrition (carotenoids, vitamin E, magnesium, and zinc) were the most significant predictors. These results are consistent with the current literature regarding the pathophysiology linking DM and OP, as well as the protective effects of dietary nutrient intake.

Gender emerged as the most significant predictor of DM-OP comorbidity, with women exhibiting a higher susceptibility. This observation aligns with epidemiological evidence indicating that postmenopausal women are at an increased risk for both conditions, primarily due to estrogen deficiency that impact glucose metabolism and bone turnover (35). Research indicates that estrogen deficiency can exacerbate insulin resistance, impairing glucose utilization and storage, which further aggravates bone loss (36). In the SWAN cohort of women, insulin resistance exhibited a biphasic relationship with bone mineral density: declining HOMA-IR slowed bone loss, whereas rising HOMA-IR accelerated it (37). Research has found that estrogen regulates vascular calcification and osteoporosis through receptor signaling, matrix proteins, and environmental-physical factors (38). BMI demonstrated a positive correlation with the risk of DM-OP comorbidity. A cohort study focusing on elderly patients with T2DM found that the prevalence of sarcopenic obesity (SO) was notably high, with significant implications for negative health outcomes, including increased risks of cardiovascular diseases and fractures (39). SHAP analysis initially identified female gender as the strongest predictor of diabetes-osteoporosis comorbidity. Subsequent stratification by both sex and age revealed nuanced risk patterns: BMI remained a dominant risk factor for women in both the 65–74 and 75–85 age groups, underscoring the persistent link between body composition and metabolic-bone health in postmenopausal women. In the oldest female subgroup (75–85 years), the influence of n–6 fatty acids and educational attainment attenuated—possibly reflecting survival bias, the predominance of non-modifiable biological factors, or cohort effects. In contrast, risk profiles in men shifted with age: BMI’s predictive importance decreased in the 75–85 year group, while lifestyle (alcohol) and sociodemographic (ethnicity) factors gained prominence. These findings highlight the need for sex- and age-specific comorbidity risk assessment to guide personalized intervention in older adults.

The identification of specific dietary nutrition as crucial protective factors offers important insights for developing intervention strategies. Carotenoids showed strong negative SHAP values, suggesting they have protective effects against the development of DM-OP comorbidities. Carotenoids, including *α*-carotene and *β*-carotene, are abundant in dark-colored vegetables and fruits, possessing potent antioxidant properties and can be metabolized in the liver into vitamin A (40). A recent dose–response meta-analysis of 13 prospective studies supports that higher dietary intake and circulating concentrations of total carotenoids, particularly *β*-carotene, are associated with a lower risk of T2DM (41). Additionally, carotenoids can reduce the risk of periodontitis (42), retinopathy (43), and cardiovascular events (44) in individuals with DM. Regarding bone health, a cross-sectional study in individuals over 50 years old showed that higher intake of β-carotene and β-cryptoxanthin is associated with a reduced risk of osteoporosis (45). Vitamin E has been identified as a significant protective factor, reinforcing its well-known antioxidant properties. A Swedish case–control study indicates that dietary vitamin E is associated with low autoantibody levels, preserved β-cell function, and reduced insulin resistance in latent autoimmune diabetes in adults (LADA), suggesting a potential protective role against autoimmune diabetes (46). A 12-week randomized, double-blind trial demonstrated that daily supplementation with 400 IU of mixed tocopherols significantly suppressed the rise in the bone resorption marker CTX in postmenopausal women with osteopenia, suggesting the potential of vitamin E supplementation in mitigating bone loss (47). Magnesium plays a vital role in glucose metabolism and the mineralization of bones. Several cross-sectional studies have found that serum magnesium levels are inversely associated with prediabetes, diabetes (48), and diabetic complications such as diabetic retinopathy (49). A recent systematic review highlighted that higher magnesium intake may support increased bone mineral density in the hip and femoral neck (50). Additionally, a cross-sectional study revealed that magnesium deficiency has a greater impact on osteoporosis than vitamin D, serving as a critical modifiable factor associated with reduced bone mineral density and increased fracture risk (51). Zinc plays a crucial protective role in insulin signaling and bone formation. A recent cohort study indicated that higher zinc levels are associated with an increased risk of T2DM in individuals with isolated impaired glucose tolerance (iIGT) (52). Zinc supplementation can correct zinc deficiency in diabetes, lower blood glucose, improve metabolic and antioxidant status, and mitigate complications such as renal, ocular, and cardiovascular issues (53). These findings underscore the potential benefits of targeted nutritional interventions that emphasize the consumption of antioxidant-rich foods and specific micronutrient supplements to enhance health outcomes in older populations.

Recent clinical trials have highlighted the promising therapeutic potential of dietary antioxidant interventions for addressing the comorbidity of DM-OP. Sun et al. (54) demonstrated that higher CDAI scores in postmenopausal women are associated with a lower risk of osteoporosis, exhibiting an age-dependent non-linear relationship. Su et al. (55) noted that the anti-resorptive agent denosumab, when combined with antioxidant therapy, can further reduce the risk of fractures. A recent review emphasized the significance of iron metabolism dysregulation in DOP, linking it to ferroptosis, proposing that therapeutic strategies targeting ferroptosis, such as the use of antioxidants, could effectively attenuate bone loss in diabetic patients (56). This dual action supports bone health in DOP. These findings collectively suggest that dietary modifications, particularly the inclusion of antioxidants play a critical role in preventing DM-OP.

The clinical significance of our findings goes beyond merely predicting risk; it also encompasses practical intervention strategies. Our model serves as a non-invasive screening tool that can identify individuals at high risk by utilizing easily accessible demographic and dietary information. Additionally, by pinpointing modifiable dietary factors, we can create targeted nutritional interventions tailored to individual needs. Furthermore, the online prediction tool we developed not only aids in the clinical implementation of these strategies but also enhances patient education about the risk factors associated with comorbidities.

Our study has several limitations that should be acknowledged. Firstly, as a fundamental limitation of this cross-sectional study, we cannot establish causality or determine the temporal sequence between dietary nutrient intake and the development of DM-OP comorbidity. The observed associations should be interpreted as correlations rather than causal effects. Secondly, our assessment of dietary intake is based on 24-h recalls, which may not accurately reflect long-term dietary patterns and are susceptible to recall bias. Lastly, the model was developed using data from the U. S. NHANES, which may limit its applicability to other populations that have different dietary habits and genetic backgrounds. Furthermore, the diagnosis of osteoporosis was based on the WHO T-score criterion (≤ − 2.5), which is derived from a reference population of young white women. While this is a widely used standard, it may not fully capture osteoporosis risk across all racial/ethnic groups and in men, as bone mineral density baselines and fracture risk relationships can differ.

Future research should focus on several key areas. First, the prospective validation of our predictive model across diverse populations; second, the exploration of optimal dietary nutrient intake thresholds for preventing comorbidities; third, the development of personalized dietary intervention protocols tailored to individual risk profiles; fourth, the incorporation of genetic and metabolomic data to improve prediction accuracy; and finally, the assessment of the model’s clinical utility in real-world healthcare environments.

## 5 Conclusion

In conclusion, our machine learning approach effectively identified significant dietary nutritional factors linked to the risk of DM-OP comorbidity in older adults. The RF model exhibited outstanding predictive performance, highlighting gender, BMI, carotenoids, vitamin E, magnesium, and zinc as the most influential predictors. These results lay the groundwork for creating targeted nutritional interventions and clinical decision support tools aimed at managing comorbidity risk in aging populations.

## Data Availability

Publicly available datasets were analyzed in this study. This data can be found at: https://wwwn.cdc.gov/nchs/nhanes/Default.aspx.

## Glossary

SHAP: SHapley Additive exPlanations
NHANES: National Health and Nutrition Examination Survey
DM: diabetes mellitus
T2DM: Type 2 diabetes mellitus
OP: osteoporosis
BMI: Body mass index
DXA: dual-energy X-ray absorptiometry
MET: metabolic equivalent of task
HbA1c: glycosylated hemoglobin
PIR: poverty income ratio
25-(OH)D: 25-hydroxyvitamin D
MUFA: monounsaturated fatty acids
PUFA: polyunsaturated fatty acids
CDAI: Composite Dietary Antioxidant Index
DII: Dietary Inflammatory Index
HEI-2020: Healthy Eating Index 2020
DASH: Dietary Approaches to Stop Hypertension
OBS: Oxidative Balance Score
SMOTE: synthetic minority oversampling technique
MLP: multilayer perceptron
SVM-RBF: support vector machine with radial basis function
AUC: area under the ROC curve
AUPRC: area under the precision-recall curve
ANOVA: analysis of variance
NRMSE: normalized root mean square error
PFC: proportion of falsely classified
ROC: Receiver Operating Characteristic
