# Supplementary material for: Multidimensional dietary assessment and interpretable machine learning models predict the risk of prediabetes/diabetes and osteoporosis comorbidity in older adults
Source: Front Nutr. 2025 Nov 17;12:1666477. doi: 10.3389/fnut.2025.1666477 (PMC12667436; doi:10.3389/fnut.2025.1666477)
Supplement: Supplementary file 1 [file Data_Sheet_1.pdf]

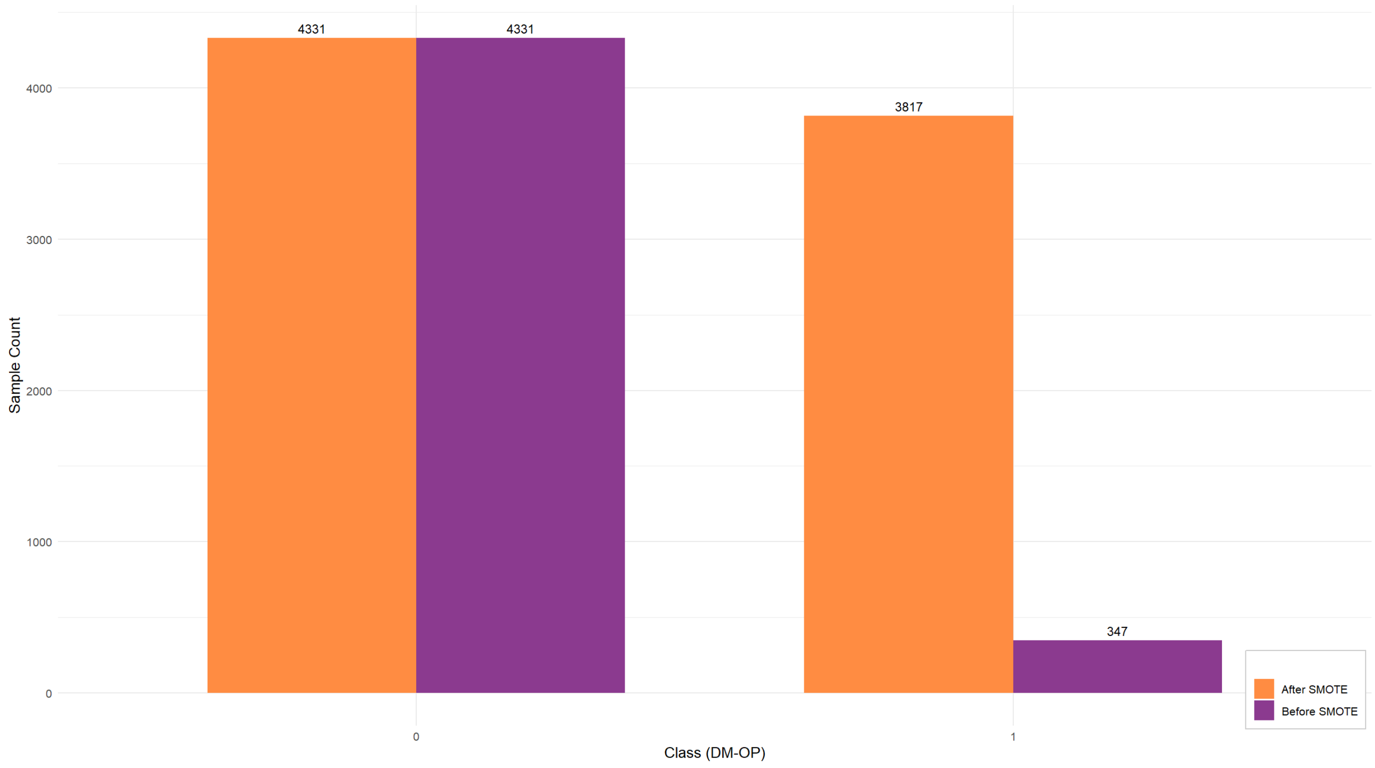

Supplementary Figure 1. Class distribution before and after SMOTE oversampling for DM − OP prediction. *The number of samples in each class (DM − OP = 1, non − DM − OP = 0) is shown before and after applying the Synthetic Minority Over-sampling Technique (SMOTE). SMOTE effectively balanced the dataset by generating synthetic samples for the minority class, reducing class imbalance and improving model training stability.*
