# Supplementary figures and images for: Multidimensional dietary assessment and interpretable machine learning models predict the risk of prediabetes/diabetes and osteoporosis comorbidity in older adults
Source: Front Nutr. 2025 Nov 17;12:1666477. doi: 10.3389/fnut.2025.1666477 (PMC12667436; doi:10.3389/fnut.2025.1666477)

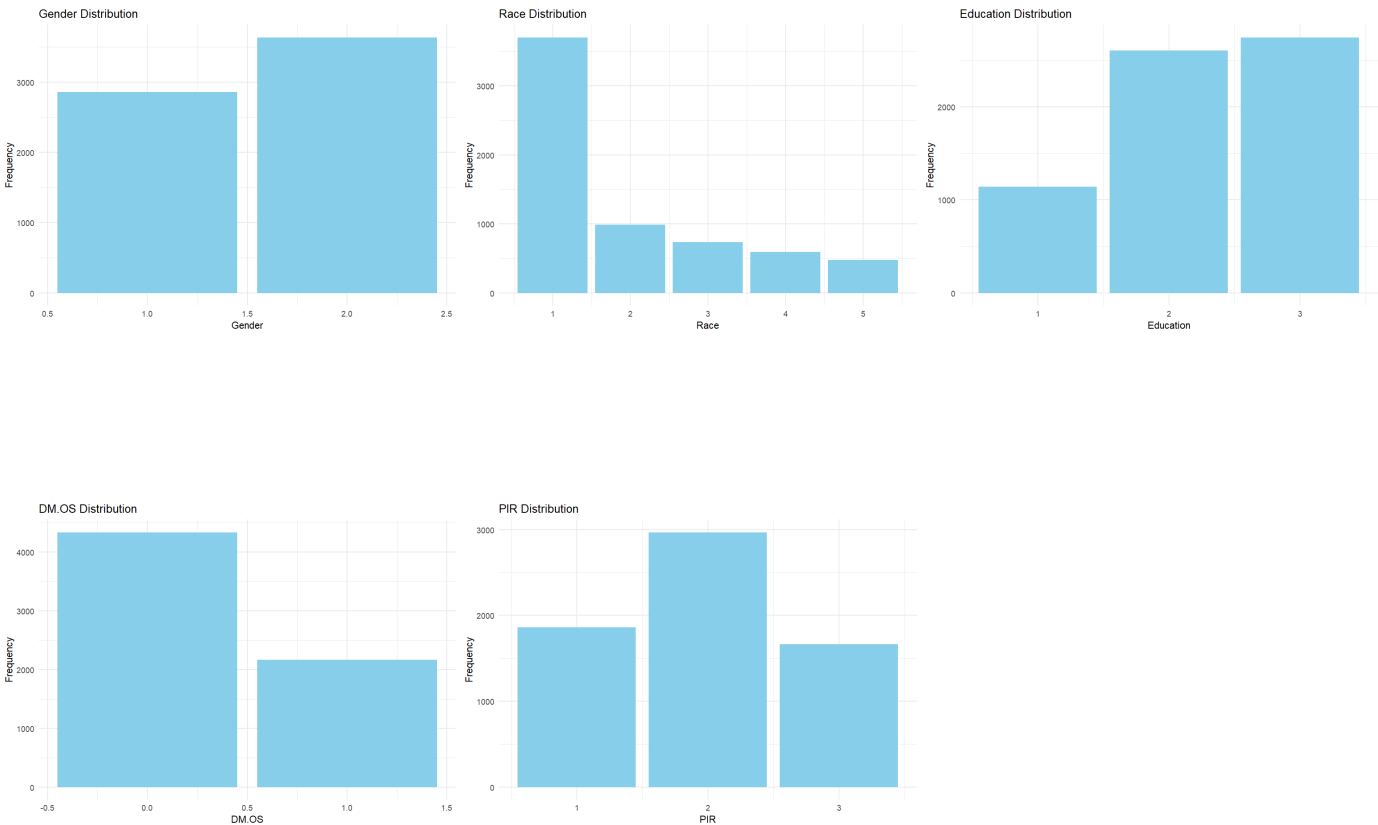

**Supplementary Figure 2. Distribution Characteristics of Categorical Variables**

Supplement: Supplementary file 2 [file Data_Sheet_2.pdf]

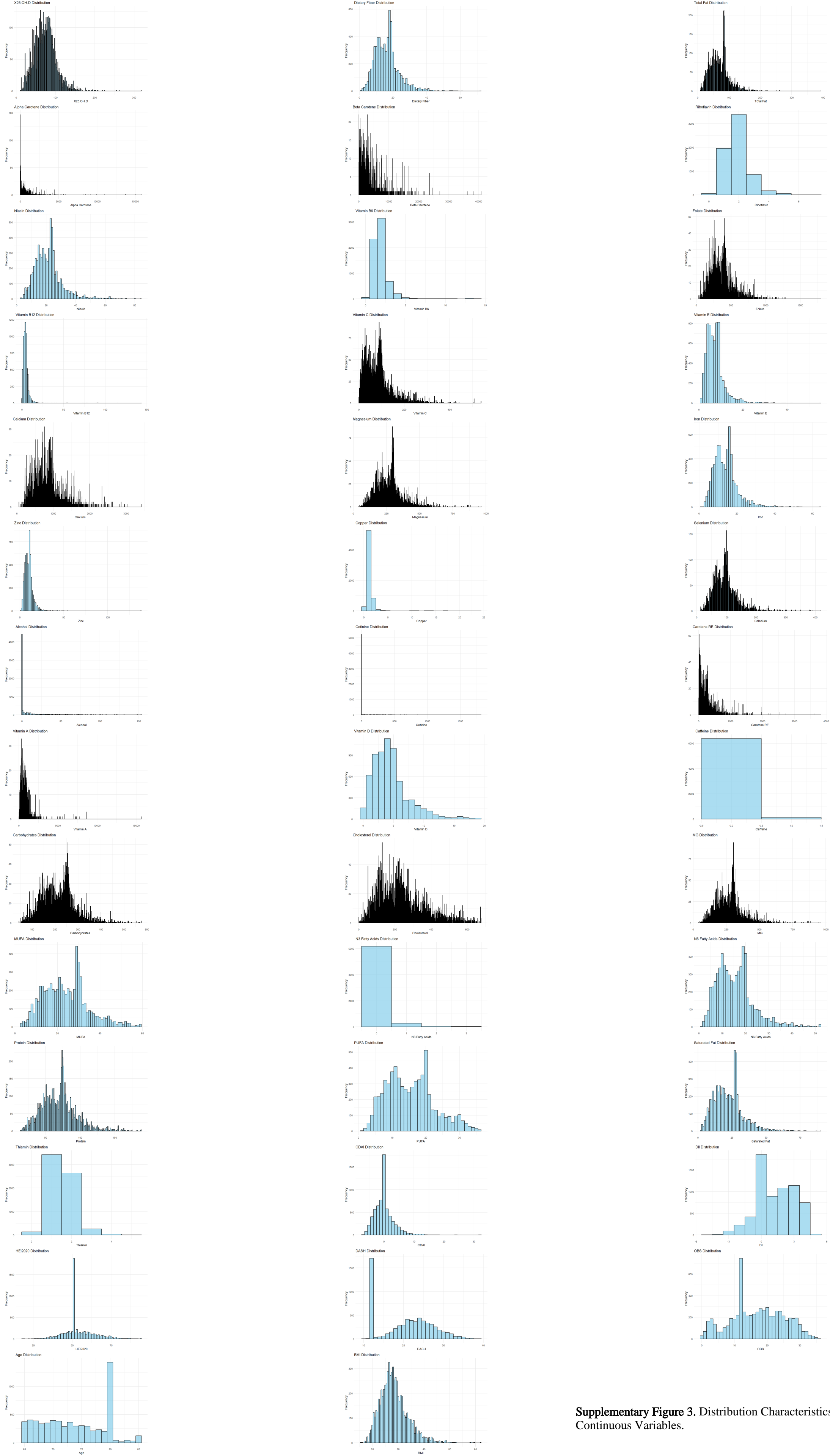

**Supplementary Figure 3.** Distribution Characteristics of Continuous Variables.

Supplement: Supplementary file 3 [file Data_Sheet_3.pdf]

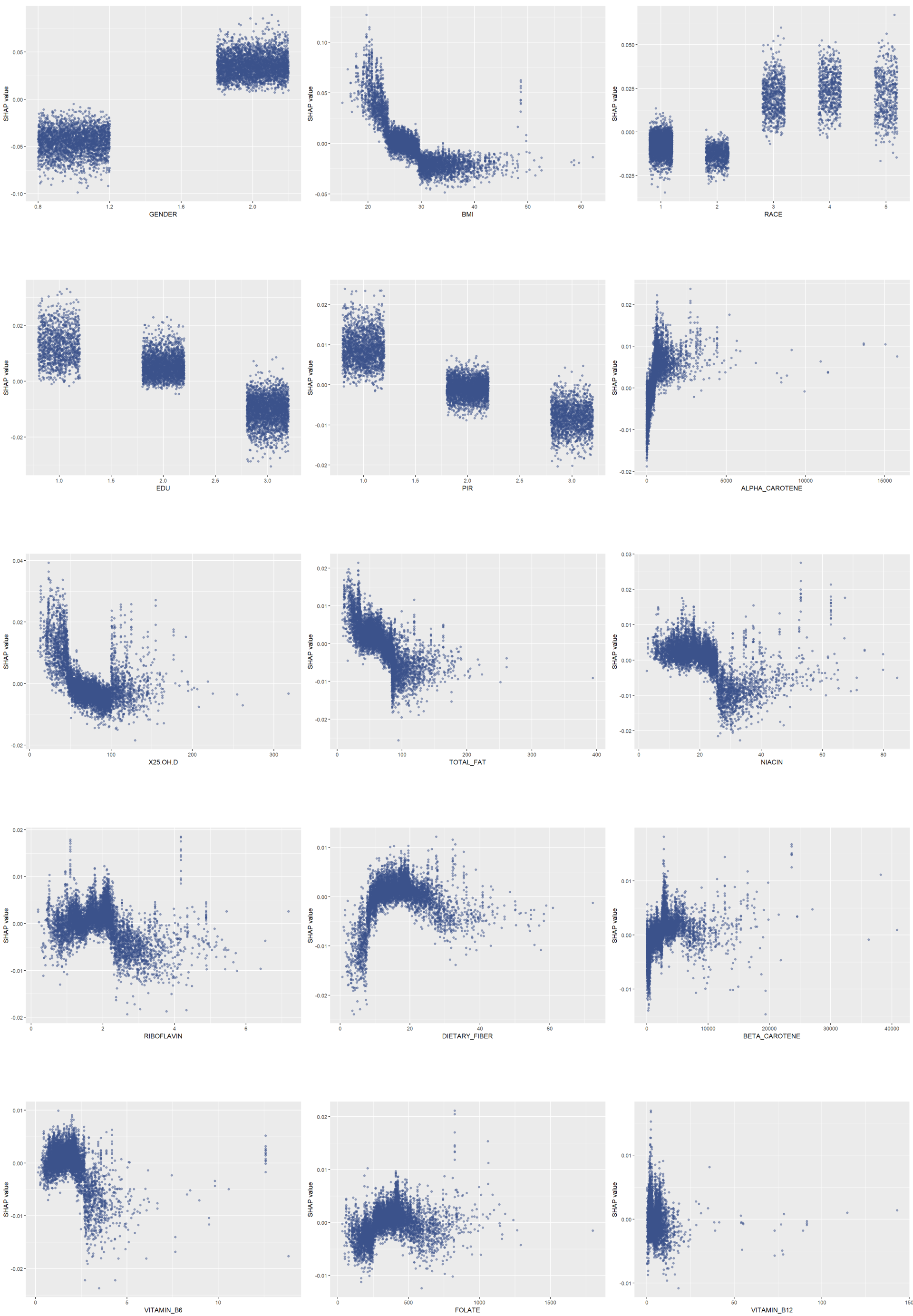

**Supplementary Figure 4. SHAP Univariate Dependency Plot.**

Supplement: Supplementary file 4 [file Data_Sheet_4.pdf]

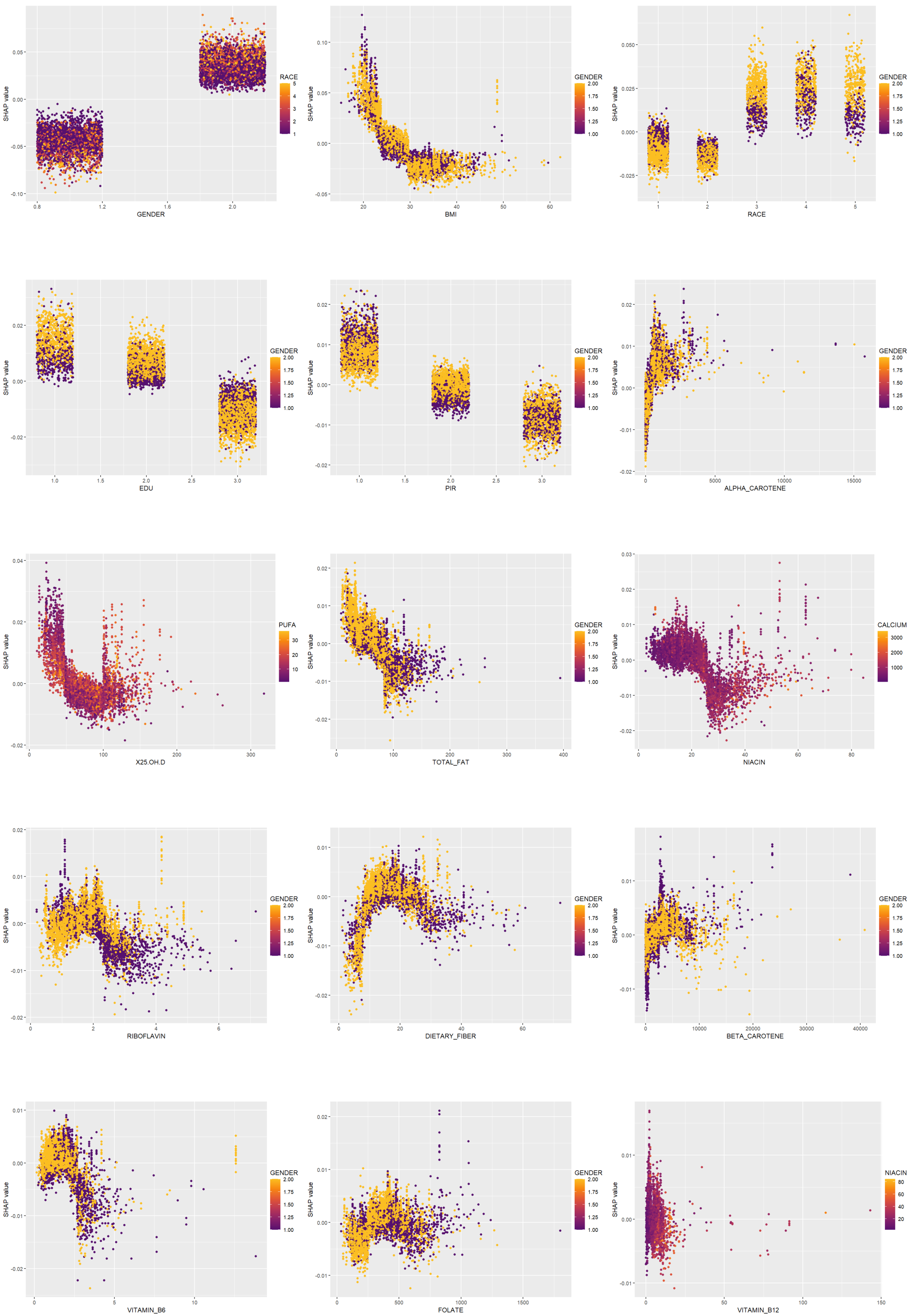

**Supplementary Figure 5. SHAP Strongest Feature Dependency Plot.**

Supplement: Supplementary file 5 [file Data_Sheet_5.pdf]
