# Supplementary material for: Multidimensional dietary assessment and interpretable machine learning models predict the risk of prediabetes/diabetes and osteoporosis comorbidity in older adults
Source: Front Nutr. 2025 Nov 17;12:1666477. doi: 10.3389/fnut.2025.1666477 (PMC12667436; doi:10.3389/fnut.2025.1666477)
Supplement: Supplementary file 6 [file Data_Sheet_6.pdf]

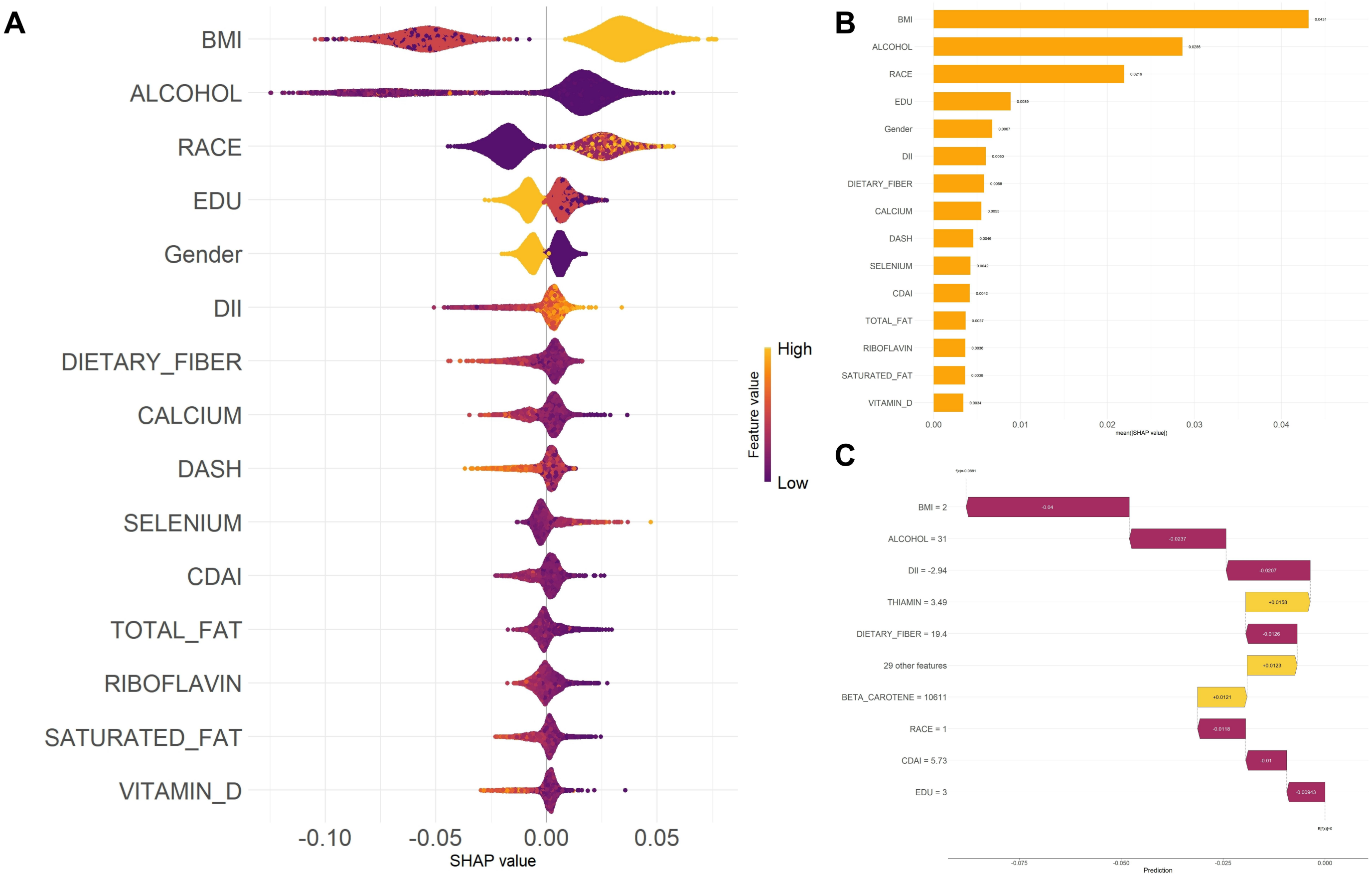

Supplementary Figure 6. Importance of Diabetes Characteristics and Interpretation of SHAP Values. *(A) SHAP beeswarm plot visualizing the distribution of SHapley Additive exPlanations (SHAP) values for each feature, colored by feature values (high = orange; low = purple). SHAP values reflect the direction (positive/negative) and magnitude of feature impact on model predictions. (B) Bar chart ranking features by their contribution to model performance; taller bars indicate greater influence on predictions. (C) SHAP waterfall plot decomposing the cumulative effect of features for a single prediction, where each bar represents the positive (yellow) or negative (purple) contribution of a feature to the final model output.*
