# Supplementary material for: Multidimensional dietary assessment and interpretable machine learning models predict the risk of prediabetes/diabetes and osteoporosis comorbidity in older adults
Source: Front Nutr. 2025 Nov 17;12:1666477. doi: 10.3389/fnut.2025.1666477 (PMC12667436; doi:10.3389/fnut.2025.1666477)
Supplement: Supplementary file 8 [file Table_1.docx]

**Supplementary Table S1：**Detailed Calculation Methods for Dietary Quality Indices

1. Composite Dietary Antioxidant Index (CDAI)^1^

| **Component** | **Details** |
| --- | --- |
| Formula | CDAI = Σ(xi - μi)/Si |
| Variables | xi = daily intake of each antioxidant for individual i<br>μi = sex-specific mean intake for each antioxidant<br>Si = sex-specific standard deviation for each antioxidant |
| Antioxidant Components | Vitamin A (μg RE/day), Vitamin C (mg/day), Vitamin E (mg/day), Zinc (mg/day), Selenium (μg/day), Carotenoids (μg/day) - sum of α-carotene, β-carotene, and carotene RE |
| Interpretation | Higher scores = greater dietary antioxidant capacity<br>Standardized: mean = 0, SD = 1 <br>Positive scores = above-average antioxidant intake |

2. Dietary Inflammatory Index (DII)^2^

| **Step** | **Process** | **Formula/Description** |
| --- | --- | --- |
| 1 | Standardization | Z-score = (Individual intake - Global mean) / Global standard deviation |
| 2 | Percentile conversion | Z-scores converted to percentile ranks |
| 3 | Centering | Centered percentile = (Percentile × 2) - 1 |
| 4 | Inflammatory scoring | Each centered percentile × inflammatory effect score |
| 5 | Summation | Sum all food parameter-specific DII scores |
| Food Parameters | 45 total parameters | Macronutrients, micronutrients, flavonoids, individual foods |
| Score Range | Theoretical range | -8.87 to +7.98 |
| Interpretation | Negative scores | Anti-inflammatory diets |
|  | Positive scores | Pro-inflammatory diets |

1. Healthy Eating Index 2020 (HEI-2020) ^3^

| **Component** | **Type** | **Maximum Points** | **Scoring Criteria** |
| --- | --- | --- | --- |
| Total Fruits | Adequacy | 5 | Higher intake = higher score |
| Whole Fruits | Adequacy | 5 | Higher intake = higher score |
| Total Vegetables | Adequacy | 5 | Higher intake = higher score |
| Greens and Beans | Adequacy | 5 | Higher intake = higher score |
| Whole Grains | Adequacy | 10 | Higher intake = higher score |
| Dairy | Adequacy | 10 | Higher intake = higher score |
| Total Protein Foods | Adequacy | 5 | Higher intake = higher score |
| Seafood and Plant Proteins | Adequacy | 5 | Higher intake = higher score |
| Fatty Acids | Adequacy | 10 | Higher unsaturated:saturated ratio = higher score |
| Refined Grains | Moderation | 10 | Lower intake = higher score |
| Sodium | Moderation | 10 | Lower intake = higher score |
| Added Sugars | Moderation | 10 | Lower intake = higher score |
| Saturated Fats | Moderation | 10 | Lower intake = higher score |

**HEI-2020 Score Interpretation**

| **Score Range** | **Diet Quality** |
| --- | --- |
| ≥80 | Good |
| 51-79 | Needs improvement |
| ≤50 | Poor |

4. Dietary Approaches to Stop Hypertension (DASH) Score ^4^

| Component | Type | Scoring Method | Points |
| --- | --- | --- | --- |
| Fruits | Beneficial | Higher intake = higher score | 1-5 |
| Vegetables | Beneficial | Higher intake = higher score | 1-5 |
| Nuts and legumes | Beneficial | Higher intake = higher score | 1-5 |
| Low-fat dairy | Beneficial | Higher intake = higher score | 1-5 |
| Whole grains | Beneficial | Higher intake = higher score | 1-5 |
| Sodium | Detrimental | Lower intake = higher score | 1-5 |
| Red and processed meats | Detrimental | Lower intake = higher score | 1-5 |
| Sweetened beverages | Detrimental | Lower intake = higher score | 1-5 |

**DASH Scoring Method**

| **Quintile** | **Beneficial Components** | **Detrimental Components** |
| --- | --- | --- |
| Q1 (lowest) | 1 point | 5 points |
| Q2 | 2 points | 4 points |
| Q3 | 3 points | 3 points |
| Q4 | 4 points | 2 points |
| Q5 (highest) | 5 points | 1 point |

5. Oxidative Balance Score (OBS) ^5^

| Component Category | Components | Scoring Direction |
| --- | --- | --- |
| Dietary Antioxidants | Vitamin A, Vitamin C, Vitamin E, α-carotene, β-carotene, lycopene, Folate, selenium, zinc, Flavonoids (when available) | Higher intake = higher score |
| Dietary Pro- oxidants | Iron, Polyunsaturated fatty acids, Total fat | Lower intake = higher score |
| Lifestyle Antioxidants | Physical activity | Higher activity = higher score |
| Lifestyle Pro-oxidants | Smoking status, Alcohol consumption | Non-smokers = highest score  Lower consumption = higher score |

**OBS Scoring Method**

| Component Type | Categorization | Scoring |
| --- | --- | --- |
| Antioxidants | Tertiles/Quartiles | Highest tertile/quartile = highest score |
| Pro-oxidants | Tertiles/Quartiles | Lowest tertile/quartile = highest score |
| Final Score | Sum of all components | Higher OBS = greater antioxidant balance |

**Supplementary Table S2:** Detailed Codebook for NOVA Food Processing Classification Applied to NHANES Food Items

| NHANES Food Code | Representative Food Description | NOVA Group | Rationale for Classification |
| --- | --- | --- | --- |
| 11101000 | Apple, raw, not specified as to source | 1. Unprocessed | Whole, fresh fruit with no added substances. |
| 32102010 | Butter, without salt | 2. Processed Culinary Ingredient | Substance derived from milk (cream) by churning, used in cooking. |
| 53203010 | Whole wheat bread, commercially prepared | 3. Processed | Made with flour, yeast, water, salt; involves baking. |
| 66208110 | Carbonated soda, cola-flavored | 4. Ultra-processed | Industrial formulation with additives like sweeteners, flavors, and preservatives. |
| ... | ... | ... | ... |

Food items were categorized using the NOVA classification system into four groups: (1) unprocessed or minimally processed foods, (2) processed culinary ingredients, (3) processed foods, and (4) ultra-processed foods (UPFs) [Citation, e.g., Monteiro et al., 2019], with daily consumption calculated in grams.

The classification was operationalized by linking the USDA food codes from the 24-hour dietary recalls to the NOVA categories. This mapping was performed by two independent research staff members trained in nutritional science, based on the detailed criteria of the NOVA system and adapted from previous studies that applied NOVA to NHANES data.

To ensure classification consistency and reliability, inter-rater agreement was assessed prior to the full analysis. Both coders independently classified a random sample of 500 food items. The agreement was evaluated using Cohen's kappa (κ) statistic, which demonstrated excellent consistency (κ = 0.85). Any coding discrepancies during the main analysis were resolved through consensus or, if necessary, adjudication by a senior investigator.

**Supplementary Table 3.** Diagnostic criteria for osteoporosis.

| Osteoporosis is a disease characterized by weakened bones and reduced bone density, making fractures more likely. Diagnosis typically involves assessing bone mineral density (BMD) using methods such as Dual-energy X-ray Absorptiometry (DXA), which measures BMD in areas like the spine, hip, and wrist. According to WHO standards, BMD is expressed as a T-score, comparing an individual's BMD to that of healthy young adults. A T-score of ≥ -1.0 indicates normal bone density, -2.5 < T-score < -1.0 suggests low bone mass (osteopenia), and T-score ≤ -2.5 diagnoses osteoporosis. In cases of fragility fractures, osteoporosis may be diagnosed even if BMD results do not meet these criteria. Beyond BMD, physicians evaluate risk factors like age, sex, family history, lifestyle (smoking, alcohol, inactivity), nutrition (calcium and vitamin D intake), and conditions or medications affecting bone metabolism^6^. |
| --- |

**Supplementary Table 4.** Brier score, calibration parameters, and decision threshold analysis of machine learning models for dysglycemia-osteoporosis comorbidity screening.

| Model | Brier Score | Calibration Intercept | Calibration Slope | Decision Threshold | PPV | NPV | Intended Use | Clinical Priority |
| --- | --- | --- | --- | --- | --- | --- | --- | --- |
| RF | 0.094 | -0.01 | 0.99 | 0.35 | 0.65 | 0.93 | Screening | First Choice |
| XGBoost | 0.108 | -0.02 | 1.03 | 0.35 | 0.60 | 0.91 | Screening | Alternative |
| KNN | 0.102 | -0.01 | 1.01 | 0.35 | 0.62 | 0.91 | Screening | Alternative |
| MLP | 0.118 | 0.01 | 0.97 | 0.40 | 0.58 | 0.89 | Triage | Alternative |
| SVM | 0.120 | -0.03 | 1.05 | 0.45 | 0.57 | 0.88 | Triage | Alternative |
| LR | 0.137 | 0.03 | 0.89 | 0.40 | 0.54 | 0.84 | Triage | Alternative |
| NB | 0.142 | 0.07 | 0.88 | 0.40 | 0.51 | 0.82 | Exploratory | Not Recommended |
| DT | 0.152 | 0.05 | 0.82 | 0.45 | 0.49 | 0.80 | Exploratory | Not Recommended |

Abbreviations: XGBoost, extreme gradient boosting tree; DT, Decision Tree; LR, Logistic Regression; MLP, Multilayer Perceptron; NB, Naive Bayes; KNN, k-Nearest Neighbors; RF, Random Forest; SVM-RBF, Support Vector Machine with RBF kernel.

**References:**

1. Composite dietary antioxidant index of antioxidant vitamins and sarcopenia risk: insights from the UK biobank and NHANES cohorts | Nutrition & Metabolism. https://link.springer.com/article/10.1186/s12986-025-00945-w.

2. Shivappa, N., Steck, S. E., Hurley, T. G., Hussey, J. R. & Hébert, J. R. Designing and developing a literature-derived, population-based dietary inflammatory index. *Public Health Nutr.* **17**, 1689–1696 (2014).

3. Healthy Eating Index-2020: Review and Update Process to Reflect the Dietary Guidelines for Americans, 2020-2025. *J. Acad. Nutr. Diet.* **123**, 1280–1288 (2023).

4. Liang, H. *et al.* Dietary Approaches to Stop Hypertension (DASH) Score and Its Association with Sleep Quality in a National Survey of Middle-Aged and Older Men and Women. *Nutrients* **12**, 1510 (2020).

5. Hernández-Ruiz, Á. *et al.* Oxidative Balance Scores (OBSs) Integrating Nutrient, Food and Lifestyle Dimensions: Development of the NutrientL-OBS and FoodL-OBS. *Antioxidants* **11**, 300 (2022).

5. Looker, A.C., et al., Prevalence of low femoral bone density in older U.S. adults from NHANES III. J Bone Miner Res, 1997. 12(11): p. 1761-8.
